# Lemon Juice Activity Against Caprine Alphaherpesvirus-1: An In Vitro Study

**DOI:** 10.3390/antibiotics15030295

**Published:** 2026-03-14

**Authors:** Francesco Pellegrini, Gianvito Lanave, Cristiana Catella, Vanessa Bachmann, Marinella Dibari, Maria Tempesta, Vito Martella, Nicola Decaro, Claudia Maria Trombetta, Michele Camero

**Affiliations:** 1 Department of Veterinary Medicine, University of Bari Aldo Moro, 70010 Valenzano, Italy; francesco.pellegrini@uniba.it (F.P.); gianvito.lanave@uniba.it (G.L.); cristiana.catella@uniba.it (C.C.); vanessa.bachmann@uniba.it (V.B.); marinella.dibari@uniba.it (M.D.); maria.tempesta@uniba.it (M.T.); vito.martella@uniba.it (V.M.); nicola.decaro@uniba.it (N.D.); 2 Department of Molecular Medicine and Development, University of Siena, 53100 Siena, Italy; trombetta@unisi.it

**Keywords:** caprine herpesvirus 1, lemon juice, antiviral, virucidal, herpesviridae

## Abstract

Caprine herpesvirus 1 (CpHV-1) is responsible for significant economic losses in goat farming. The CpHV-1 genital infection in goats has been used as a homologous animal model for the study of human herpes simplex virus type 2 (HSV-2). This study aimed to investigate the in vitro virucidal and antiviral effect of lemon juice (LJ) and its main component, citric acid (CA), against CpHV-1 on Madin-Darby Bovine Kidney (MDBK) cells. Cytotoxicity was assessed using an XTT assay, while viral titers were determined by the Reed–Muench method and viral DNA was quantified via qPCR. Pure LJ (pH 2.3) and its corresponding CA solution demonstrated potent and rapid virucidal activity, reducing the viral titer by over 5.0 log10 TCID_50_/50 µL within 1 min. When applied after viral entry, a non-cytotoxic dilution of LJ (pH 4.32) significantly inhibited viral replication, causing a 2.5 log10 TCID_50_/50 µL reduction in viral titer and a corresponding decrease in viral DNA. The antiviral effects were minimal at a near-neutral pH of 6.67, probably interacting with envelope structures. These results suggest that LJ could be a potential low-cost topical agent or disinfectant for controlling CpHV-1 in goat populations and offer a basis for translational research on human herpesviruses.

## 1. Introduction

Caprine herpesvirus 1 (CpHV-1) belongs to the *Orthoherpesviridae* family, subfamily *Alphaherpesvirinae*. This enveloped DNA virus is characterized by rapid, lytic growth cycles in the genital tract. CpHV-1 is widespread in the goat population worldwide, with a seroprevalence of 30–40% in Mediterranean countries [1]. In adult goats, CpHV-1 is responsible for severe and recurrent infections with lesions on the vulvar and genital mucosa, characterized by vesicles, ulcers, and scabs [2].

Although CpHV-1 has been associated with abortion in goats, the behavior of the virus in the evolution of pregnancy has not been clarified yet [3]. CpHV-1 is highly prevalent in countries where goat farming represents a significant source of income for farmers. CpHV-1 has a negative economic impact on goat breeding, being responsible for failed insemination, decreased milk production, abortions, and neonatal mortality [1].

Several antiviral agents have been tested in vitro and in vivo against CpHV-1 [4,5,6,7]. Herpesvirus resistance to antiviral drugs primarily arises via mutations in viral thymidine kinase (TK) or DNA polymerase (Pol), reducing drug activation or binding and thereby requiring alternative therapies [8,9]. In recent years, a growing interest in naturally derived molecules with antiviral activity has been reported. These molecules represent a possible alternative in cases of resistance and/or ineffectiveness to conventional drugs and could supplement pharmacological therapy [10,11,12]. CpHV-1 shares many similarities with the human herpes simplex type 2 (HSV-2), including the preferential tropism for the genital tract, the sequela of the evolution of skin lesions, the route of transmission and latency in the sacral ganglia [2,13]. For these reasons, the goat is considered an excellent animal model for studying HSV-2 infection and for evaluating the efficacy of antiviral molecules for the treatment of genital herpes in humans [4,14]. An estimated 205 million people aged 15–49 (5.3%) experienced at least one symptomatic episode of genital herpes in 2020 [15].

Numerous synthetic [16,17] and natural [18] antiviral substances have been tested against HSV-1 and 2 [19,20]. Recent studies indicate that plants and edible fruits are a valuable source of antiviral compounds, prompting further essential research to confirm scientific data [21]. Fruit extracts, particularly from citrus, possess antiviral properties due to the presence of compounds such as hesperidin and other flavonoids, which appear to inhibit the virus’ ability to infect cells [22,23,24,25]. Lemon juice, rich in vitamin C, helps prevent infections and has been traditionally used to treat scurvy, sore throat, fever, rheumatism, hypertension, and chest pain [26,27]. Like other citrus fruits, lemon juice contains significant concentrations of citric acid in all varieties (approximately 47 g/L), which is thought to be the main component responsible for its antiviral activity [28].

The potential virucidal mechanism for organic acids likely depends on their ability to permeate the viral envelope or capsid and damage the viral genome [29]. The effect of citric and acetic acid on the inactivation of human norovirus surrogates has been previously examined [30,31]. In Africa, lime and lemon juices have been used as contraceptives and for vaginal hygiene; nevertheless, the use of these pure, undiluted juices for such purposes has not been widely recommended [32].

In the present study, the virucidal and antiviral properties of LJ were evaluated as a natural mixture of citric acid, flavonoids, ascorbic acid and other components against CpHV-1.

## 2. Materials and Methods

### 2.1. Lemon Juice (LJ)

Lemon juice (LJ) was extracted by squeezing the pulp of lemons of the “four-season” variety purchased from a local grocery store. The raw juice was centrifuged at 3000 rpm for 10 min in a refrigerated centrifuge (+4 °C), and the supernatant was subsequently filtered through a 0.22 µm pore size membrane filter (Merck KGaA, Darmstadt, Germany). The pH of the pure juice and subsequent dilutions was determined using a digital pH-meter, calibrated prior to use. All measurements were repeated in triplicate.

### 2.2. Citric Acid (CA)

To evaluate the specific effect of citric acid (CA) (Merck KGaA, Darmstadt, Germany), we used solutions with pH levels matching those of undiluted (pH 2.3) and diluted LJ (pH 4.32 and 6.67). To distinguish between the acidity effect of and the potential effect of the citrate moiety [30], all solutions were realized with the same molar concentration (0.24 mol/L). This concentration corresponds to the average CA level (47 g/L) reported in LJ [28]. The pH 2.3 solution was prepared by dissolving CA in sterile water and adjusting the pH with a NaOH solution. The pH 4.32 and 6.67 solutions were prepared by mixing the 0.24 M CA solution with a suitable volume of trisodium citrate, also at the same concentration.

### 2.3. Cells and Viruses

All in vitro assays were performed using Madin-Darby Bovine Kidney (MDBK) cells (Biogenetics Diagnostic S.r.l., Ponte San Nicolò, Italy) cultured at 37 °C in the presence of 5% CO_2_. The cells were maintained in Dulbecco-MEM (D-MEM) (Corning ^®^, Glendale, AZ, USA) and supplemented with 10% fetal bovine serum, 100 IU/mL penicillin, 0.1 mg/mL streptomycin, and 2 mM l-glutamine (Corning ^®^, Glendale, AZ, USA). The same medium was used for antiviral tests. The Ba.1 CpHV-1 field strain [33] was cultured and titrated on MDBK cells. The resulting viral stocks, with a titer of 10^6.50^ Tissue Culture Infectious Dose (TCID_50_)/50 μL, were preserved at −80 °C prior to their use in all experiments.

### 2.4. Cytotoxicity Assay

Cellular viability following exposure to both LJ and CA was measured via a colorimetric in vitro toxicology assay kit (Sigma-Aldrich Srl, Milan, Italy) based on 3-(4,5-dimethylthiazol-2-yl)-2,5-diphenyltetrazolium bromide (XTT). Viability was determined by measuring the absorbance signal with a spectrophotometer using a 450 nm wavelength (Bio-Rad Laboratories, Inc., Segrate, Italy). The assay was conducted as previously described [34]. Briefly, Confluent 96-well plates containing 24 h confluent MDBK cells (D-MEM cultured) were treated with several LJ dilutions (1:20, 1:200, 1:2000, 1:20,000, and 1:200,000) and with corresponding CA controls, adjusted to match the pH of the corresponding LJ dilutions. The percentage of cytotoxicity was calculated using the following formula:

%Cytotoxicity=ODcontrol cells/(ODcontrol cells−ODtreated cells)×100

The non-cytotoxic threshold was determined by identifying the concentration at which 80% of treated MDBK cells remained viable (CC_20_), when compared to untreated ones (control cells) [11,35]. All experiments were performed in triplicate.

### 2.5. Virucidal Activity Assay LJ

The potential virucidal effect of LJ against CpHV-1 was evaluated by direct exposure of the virus to pure juice (pH 2.3) and to a 1:2000 diluted solution, below the cytotoxic threshold (pH 6.67). One hundred microliters of the stock virus solution were mixed with 900 µL of either pure juice or diluted solution at room temperature. After 30 s, 1, 3, 5, 15, and 30 min, the virus mixed with either LJ or CA, along with the control (virus mixed with D-MEM), was collected and subjected to viral titration on MDBK cells [11,36]. To ensure the neutralization of the acidic effect, the mixtures were immediately exposed to serial 10-fold dilutions in D-MEM and pH was measured to ensure the stopping of the residual virucidal activity. The experiments were performed in triplicate.

### 2.6. Virucidal Activity Assay CA

To evaluate the virucidal kinetics, 900 µL of CA solution at pH 2.3 and 6.67 were mixed with a 100 µL aliquot of the viral stock suspension. At designated contact intervals (30 s, 1, 3, 5, 15 and 30 min), mixtures of CA virus and D-MEM/virus (control virus) were collected and subjected to viral titration to MDBK [36]. To ensure the neutralization of the acidic effect, the mixtures were immediately subjected to serial 10-fold dilutions in D-MEM and pH was measured to ensure the stopping of the residual virucidal activity. The experiments were performed in triplicate.

### 2.7. Antiviral Activity Assay

Antiviral activity against CpHV-1 was evaluated using LJ diluted at the maximum non-cytotoxic dose (1:200 dilution, pH 4.32) and below the cytotoxic threshold (1:2000 dilution, pH 6.67). In order to better identify the phase of viral inhibition by LJ, two different protocols (A and B) were set up. All experiments using CA were performed with solutions at pH levels corresponding to those used for LJ. All experiments were performed in triplicate.

#### 2.7.1. Protocol A: Viral Infection of Cell Monolayers Before LJ Treatment

Confluent 24 h-old MDBK cell monolayers in 24-well plates were used. The cells were infected with 100 µL of CpHV-1 containing 100 TCID_50_. After 1 h of adsorption at 37 °C, the inoculum was removed, the cell monolayers were washed once with fresh non-supplemented D-MEM, and 1 mL of the 1:200 (pH 4.32) or 1:2000 LJ solution (pH 6.67) was added. At 24-, 48-, and 72 h post-treatment, aliquots of the supernatants were collected for subsequent viral titration and DNA quantification, as previously described [11].

#### 2.7.2. Protocol B: Viral Infection of Cell Monolayers After LJ Treatment

Experiments were performed using MDBK cells grown to confluence for 24 h in 24-well tissue culture plates. Prior to viral exposure, the monolayers were treated for 1 h at 37 °C with 1 mL of the 1:200 (pH 4.32) and 1:2000 LJ solutions (pH 6.67). After this pre-treatment phase and removal of the treated medium, the monolayers were washed once with fresh non-supplemented D-MEM and then inoculated with 100 µL of CpHV-1 containing 100 TCID_50_. Following 1 h of viral adsorption at 37 °C, the inoculum was removed, and the monolayers were washed with fresh D-MEM before adding 1 mL of maintenance medium (D-MEM). To monitor viral replication kinetics, supernatant aliquots were collected at 24-, 48-, and 72 h post-infection for subsequent viral titration and DNA quantification, as previously described [11].

### 2.8. Viral Titration

Ten-fold serial dilutions (up to 10^−8^) of each supernatant were titrated in quadruplicates in 96-well plates containing MDBK cells. The plates were incubated for 72 h at 37 °C in a 5% CO_2_ atmosphere. The viral titer was calculated based on the observation of cytopathic effect (CPE) using the Reed–Muench method [36,37]. The experiments were performed in triplicate.

### 2.9. Quantification of CpHV-1 DNA

Aliquots from each supernatant collected from the 24-well cell culture plates were subjected to quantitative PCR (qPCR) using CpHV-1-specific primers and probe, as previously described [11]. Amplification was performed in a 25 µL reaction volume containing: 12.5 µL of IQ™ Supermix (Bio-Rad Laboratories Srl, Segrate, Italy), 900 nM of each primer (CpHV-1 For: 5′-TACCTCTTTCCCGCGCCCACG-3′ and CpHV-1 Rev: 5′-TGTACACGCCCTCGGTCGCC-3′), 200 nM of the CpHV-1Pb probe (5′-FAM-CCGCCTGCCCCTCACCATCCGCTCC-TAMRA-3′), and 10 µL of template DNA. The thermal cycling protocol consisted of a 10 min initial activation step at 95 °C for the iTaq DNA polymerase, immediately followed by 45 cycles of denaturation at 95 °C for 1 min and combined primer annealing and extension at 70 °C for 1 min.

### 2.10. Data Analysis

Results derived from both virucidal and cytotoxicity assays, reported as the mean ± standard deviation (SD), were log_10_-transformed, and the results from the cytotoxicity assays were analyzed using non-linear curve fitting. Furthermore, a dose–response curve was generated through non-linear regression analysis to assess the goodness-of-fit. The CC_20_ was calculated from the fitted dose–response curves obtained in each experiment. The normality of the data distribution was assessed using the Shapiro–Wilk test. Group comparisons were conducted using a one-way analysis of variance (ANOVA), with Bonferroni’s correction applied as post hoc multiple comparisons. Statistical significance was set at *p* < 0.05. Statistical analyses were performed using GraphPad Prism software v10.2.1 (Dotmatics, Boston, MA, USA).

## 3. Results

### 3.1. Lemon Juice pH Measurement

The measured pH values corresponding to pure and serial dilutions (1:20, 1:200, 1:2000, 1:20,000, and 1:200,000) tested were 2.3 (pure juice), 2.32, 4.32, 6.67, 7.16 and 7.36.

### 3.2. Cytotoxicity Assay

Cytotoxicity was assessed by measuring cell viability using the XTT colorimetric assay. MDBK cells were exposed for 72 h to various dilutions of LJ (1:20, 1:200, 1:2000, 1:20,000, and 1:200,000) and their corresponding pH-matched CA solutions.

**Figure 1:**
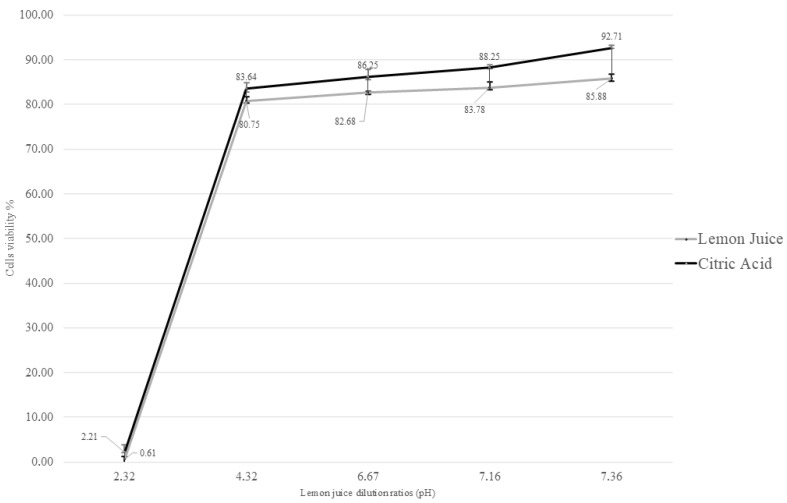
Cytotoxicity of MDBK cells treated with diluted LJ (1:20, 1:200, 1:2000, 1:20,000, and 1:200,000) and matching CA solutions at 72 h post-treatment and calculated using the XTT assay. The ANOVA model revealed a statistically significant effect between dilutions, with increased cell viability.

Based on the fitted dose–response curves, the CC_20_ value for LJ was determined to be the 1:200 dilution (pH = 4.32) (Figure 1). When comparing the cytotoxicity across all tested LJ dilutions, the ANOVA model revealed a statistically significant effect (F = 282.23, *p* < 0.01).

Pairwise comparisons showed that the cytotoxicity of the 1:20 dilution was significantly higher than that of all other dilutions (*p* < 0.01). A statistically significant decrease in cytotoxicity was also observed when comparing the 1:200 dilution to the 1:2000, 1:20,000, and 1:200,000 dilutions (*p* < 0.01). No statistically significant differences in cytotoxicity were observed among the other concentrations (*p* > 0.05).

### 3.3. Virucidal Activity Assay LJ

The virucidal effects of LJ were evaluated by exposing CpHV-1 to both undiluted juice (pH 2.3) and over the maximum non-cytotoxic dilution (1:2000, pH 6.67) at various contact times. The antimicrobial effects of a substance can often be manifested by using dosages below the maximum non-cytotoxic dose, thus reducing costs and possible toxic effects in prolonged therapies.

Treatment with undiluted LJ resulted in a powerful and rapid inactivation of the virus. A significant reduction in viral titer of 5.00 log_10_ TCID_50_/50 µL was observed after just 30 s of contact, with this reduction reaching 5.25 log_10_ after 15 min (*p* < 0.01). To determine if this effect was primarily due to acidity, a pH-matched CA solution was tested. The CA solution at pH 2.3 exerted a similar virucidal effect, causing a viral reduction of 4.75–5.00 log_10_ within the first 5 min and 5.25 log_10_ at later time points (*p* < 0.01).

**Figure 2:**
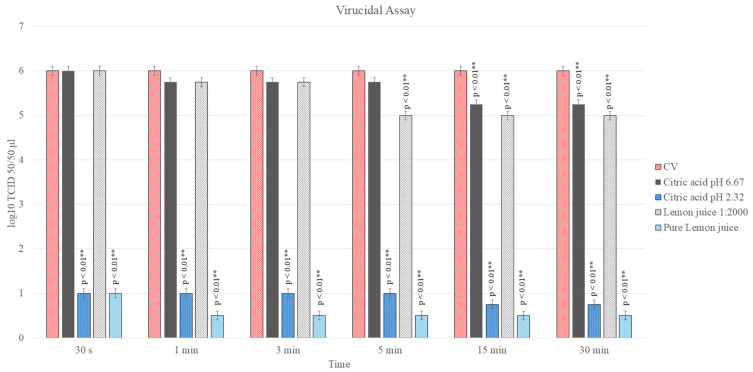
Virucidal effect of LJ and CA against CpHV-1 for 30 s, 1, 3, 5, 15 and 30 min at room temperature and subsequently titrated in MDBK. Viral titers of CpHV-1 were expressed as log_10_ TCID_50_/50 μL, and treated viral titers were compared to those of the control virus (CV). The bars in the figures indicate means. Error bars indicate standard deviation. Level of significance has been reported (*p* ≤ 0.01 **).

In contrast, virucidal activity was limited using LJ diluted over the non-cytotoxic maximum dose (1:2000, pH 6.67). Only a minor viral reduction of 0.25 log_10_ was observed within the first 5 min (*p* > 0.05). However, a more pronounced and statistically significant reduction of 1.00 log_10_ was achieved after 15 and 30 min of contact (*p* < 0.05). The corresponding CA solution at pH 6.67 displayed a similar trend, with no significant effect in the first 5 min, followed by a significant viral reduction of 0.75 log_10_ after 15 and 30 min of contact (*p* < 0.05) (Figure 2).

### 3.4. Antiviral Activity Assay

#### 3.4.1. Protocol A: Viral Infection of Cell Monolayers Before Treatment

**Figure 3:**
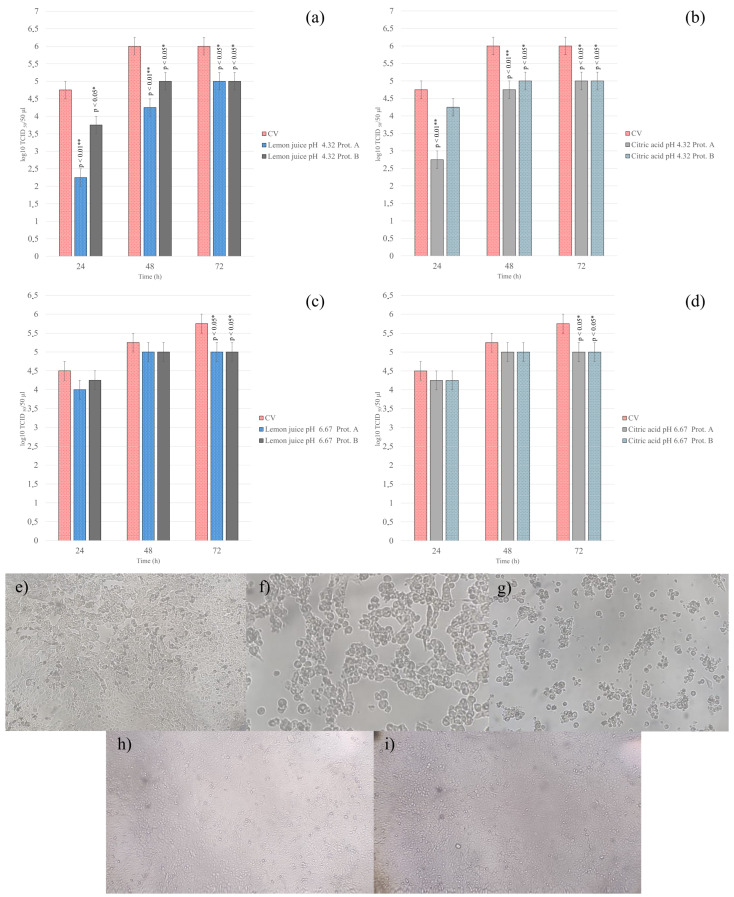
Evaluation of viral particles’ viability after infection of cell monolayers treated with LJ and CA solutions at pH 4.32 (**a**,**b**) and 6.67 (**c**,**d**). Supernatants of untreated and treated cells were collected 24, 48, and 72 h after infection. Viral titers were evaluated by the endpoint dilution method and expressed as log_10_ TCID _50_/50 μL. Error bars indicate standard deviation. Level of significance has been reported (*p* ≤ 0.05 *; *p* ≤ 0.01 **). Microscopic observation of MDBK cells showing the progression of the cytopathic effect (CPE) induced by CpHV-1 infection at 24-, 48-, and 72 h post-infection (**e**–**g**) compared to healthy uninfected control cells cultured for 24 and 72 h (**h**,**i**) (20×). The images show the characteristic rounding and detachment of cells in the infected groups versus the intact monolayer in the controls.

**Figure 4:**
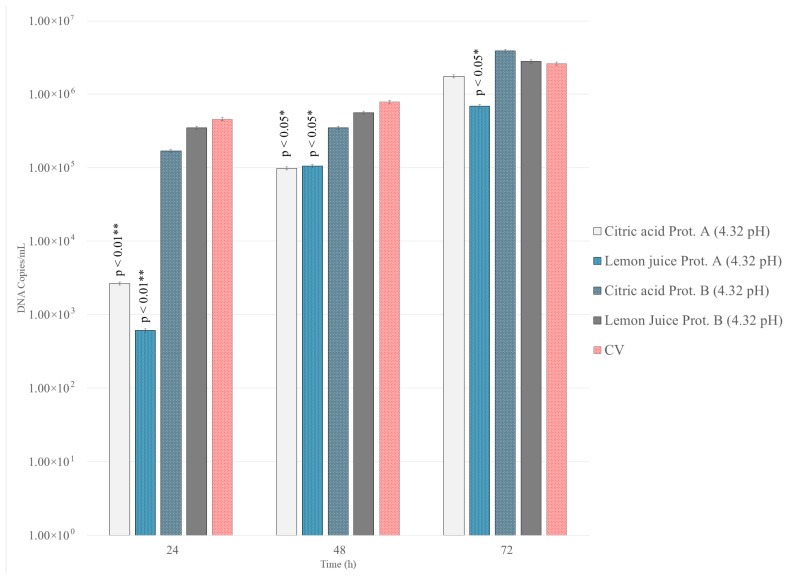
Viral infection of cell monolayers after treatment with diluted LJ (1:200) and CA at pH 4.32. Supernatants of untreated and treated cells were collected 24, 48, and 72 h after infection. Viral nucleic acids were extracted and quantified by qPCR as log_10_ viral DNA copy number/10 μL. The bars in the figures indicate means. Error bars indicate standard deviation. Level of significance has been reported (*p* ≤ 0.05 *; *p* ≤ 0.01 **).

To determine if LJ and CA could inhibit viral replication after infection was established, treatments were applied one-hour post-infection. At the non-cytotoxic pH of 4.32, both treatments demonstrated a significant antiviral effect. At 24 h post-infection, LJ (1:200, pH 4.32), significantly reduced the viral titer by 2.5 log_10_ TCID_50_/50 µL compared to untreated controls (*p* < 0.01, Figure 3a). This inhibitory effect persisted, though diminished, at 48 h (1.75 log_10_ reduction; *p* < 0.01) and 72 h (1.00 log_10_ reduction; *p* < 0.05). The CA solution (pH 4.32) showed a similar trend, with a significant viral titer reduction of 2.00, 1.25 and 1.00 log_10_ at 24, 48 and 72 h, respectively (*p* < 0.05, Figure 3b). This reduction in infectivity was supported by a significant decrease in viral DNA copy numbers for both LJ and CA treatments at this pH (*p* < 0.05) (Figure 4).

In contrast, the antiviral activity was substantially weaker when using solutions with a pH of 6.67. For both LJ and CA, only minimal and delayed reductions in viral titer were observed at 72 h post-infection (*p* < 0.05, Figure 3c,d). Moreover, neither treatment at pH 6.67 resulted in a statistically significant decrease in viral DNA copy numbers at any time point (*p* > 0.05).

#### 3.4.2. Protocol B: Viral Infection of Cell Monolayers After Treatment

Cell monolayers were pre-treated with LJ or CA solutions (pH 4.32 and 6.67) before being exposed to the virus. For LJ (1:200, pH 4.32), minimal reductions in viral titer of 1.00 log_10_ were observed at 24-, 48- and 72 h post-infection (*p* < 0.05, Figure 3a). For CA (pH 4.32), only minimal yet significant reductions in viral titer of 1.00 log10 were observed at 48- and 72 h post-infection (*p* < 0.05, Figure 3b).

Moreover, neither treatment at pH 6.67 resulted in a statistically significant decrease in viral titer at 24 and 48 h (*p* > 0.05). For both LJ and CA, minimal but significant reductions in viral titer of 0.75 log_10_ were observed at 72 h post-infection (*p* < 0.05, Figure 3c,d).

There were no statistically significant differences in either viral titer or viral DNA copy number at any time point (24-, 48-, or 72 h) between the pre-treated cells and the untreated control cells (*p* > 0.05, Figure 4).

#### 3.4.3. Statistical Analysis

A strong positive correlation was found between the reduction in CpHV-1 infectious titers and the decrease in viral DNA copies/mL, as determined by Spearman’s rank correlation analysis (r = 0.8258, *p* = 0.0003). This high degree of correlation confirms that the antiviral effect of LJ observed in cell culture is consistently reflected at the molecular level.

## 4. Discussion

Research on antiviral agents of natural origin has been receiving increasing attention not only to discover more sustainable therapies but also to scientifically validate traditionally known remedies, whose healing effects are sometimes anecdotal and unfounded. In this context, our study investigated for the first time the efficacy of LJ and its main component, CA, against CpHV-1 in vitro.

The results obtained clearly demonstrate a dual activity, comprising powerful virucidal action and a significant inhibition of viral replication. The virucidal effect was found to be extremely rapid and powerful at pH 2.3 (pure juice), causing a drastic reduction in the infectious titer in less than a minute.

Antimicrobial activity was found to be strictly pH dependent, as confirmed by the overlapping results obtained with the pH-equivalent CA solution. This suggests that the main mechanism of action is direct and irreversible damage to the structural integrity of the virion, probably through the denaturation of viral envelope proteins, which are essential for infection. For herpesviruses, exposure to low pH is a known cause of irreversible conformational changes in surface glycoproteins, which are essential for fusion with the host cell’s membrane. For glycoprotein B (gB), which is highly conserved among all herpesviruses, it has been shown that exposure of the virion to an acidic pH (pH 5.0) causes irreversible inactivation of its fusogenic properties [38,39].

Our study, in addition to the virucidal effect, showed significant antiviral activity when the treatment was applied after infection of cell monolayers (Protocol A). Again, the effect was markedly pH-dependent, with a robust inhibition of replication at pH 4.32, confirmed by both the reduction in the viral titer and the quantification of the viral DNA. At near-neutral pH (6.67), the effect was weak, with limited impact on viral replication. In contrast, pre-treatment of cells (Protocol B) demonstrated no protection against subsequent infection, indicating that the mechanism does not lie in the induction of a cellular antiviral state but rather in direct interference with the replication cycle already underway or with the stability of new viral particles. In addition, the observed antiviral activity (Protocol A) and lack of efficacy in the pre-treatment assay (Protocol B) may suggest that LJ permanently alters cell surface receptors required for viral attachment. However, significant gaps in how LJ interacts with viral particles are yet to be addressed and further studies will be needed to elucidate the precise molecular targets and exact step of inhibition.

Comparative studies on other viruses have shown that weak acids, at similar pH values, can exhibit different virucidal effects. For example, exposing the influenza A virus to a pH 4.0 acetic acid solution was effective, while citric and oxalic acids were not [40]. This suggests a different ability of the acid to penetrate the viral lipid envelope and to interact with internal components, which may be a determining factor.

In addition, LJ is not a simple citric acid solution, but a phyto-complex. Lemon essential oil, also present in minimal amounts in the juice, is rich in monoterpenes such as limonene (up to 67%), β-pinene and γ-terpinene [35,41]. Both limonene and β-pinene have been shown to possess intrinsic anti-herpetic activity against HSV-1, through direct interaction with the virion at 5.9 μg/mL and 3.5 μg/mL, respectively [42]. Although in our study the effects of these compounds may seem secondary to that exerted by low pH, it is plausible that the efficacy of lemon juice derives from a synergistic effect between extreme acidity and these naturally occurring bioactive compounds. It is important to note that the primary organic acid content, specifically citric acid, is highly conserved (approximately 47 g/L) across different lemon varieties. However, future pivotal in vitro studies will focus on the isolation and quantitative characterization of these specific classes of compounds, assessing potential variations based on specific cultivar differences, seasonality, and geographical origin. This will allow for a better standardization of the extract, preservation and evaluation of any synergistic antimicrobial properties as well as cytotoxicity levels.

Contextualizing these findings in the context of CpHV-1 pathology is critical. This virus represents a burden for farmers due to its direct economic impact, through abortions and high neonatal mortality [3]. These events have a particularly critical economic weight, especially in those countries where there is a high demand for kids due to traditions at certain times of the year, such as Easter and Christmas. Fortunately, unlike other herpesviruses, reactivation of latency by CpHV-1 is a rare event [13], which makes controlling transmission and primary infection the most logical and effective strategy. In the livestock market, the low economic value of the individual goat discourages the use of sophisticated and expensive veterinary medical therapies. Farmers, for purely economic reasons, may prefer to remove or slaughter infected animals rather than choose therapeutic treatment. These reasons, which might seem like a limitation, give practical value to our results. A treatment based on a product such as lemon juice (cheap, easy to find and safe) could be a valid practical tool. It could be used topically for skin lesion treatment or, given its powerful virucidal action, as a low-cost environmental disinfectant to reduce the viral load on surfaces and limit transmission within the flock.

The in vitro nature of this work constitutes a fundamental limitation, and further investigation will be necessary to confirm these findings in a more complex biological system, considering the host immune response or mucosal barriers. While MDBK cells provide a robust model for preliminary screening, the characteristics of this cell line may not fully replicate the physiological state of the natural target tissue, the genital mucosa. Efficacy and safety will necessarily have to be validated by in vivo studies to assess potential risks and therapeutic outcomes. Moreover, potential variations due to seasonality, specific cultivar differences and geographical origin remain to be fully explored. While we hypothesize a pH-dependent mechanism, specific molecular assays to pinpoint the exact mechanism or replication step inhibited will be pivotal before considering any practical application.

However, the importance of this research goes beyond the veterinary field. The well-known biological and pathogenetic similarity between CpHV-1 infection and Herpes Simplex Virus type 2 (HSV-2) infection in humans makes this animal model extremely valuable. Some non-hormonal contraceptive medical products possess a mechanism of action, relying on a combination of lactic acid, citric acid and potassium bitartrate to maintain vaginal pH at an acidic level (between 3.5 and 4.5) [43,44]. This acidic environment immobilizes sperm and has been shown to be active against HSV-1 and other pathogens in preclinical studies, acting as a potential microbicide [45,46].

Therefore, the demonstration that a simple vegetable product, such as LJ, can effectively inactivate an animal herpesvirus opens interesting perspectives for translational research, suggesting new hypotheses for the development of low-cost topical microbicides or therapeutic adjuvants. The traditional, but strongly discouraged, use of lemon or lime juice as vaginal lavage for contraceptive or hygienic purposes, especially in some regions of Africa, is documented in the literature [47,48]. Application of lime juice at high concentrations (50% to 100%) causes cervical dysplasia, pain, and genital irritation in a significant proportion of women [48]. Even at lower concentrations (e.g., 20%), although no serious damage is observed, side effects such as irritation and dryness have been frequently reported [32].

Given the aforementioned low economic value of individual animals in the livestock market, often discouraging expensive veterinary therapies, LJ represents a cheap and easy-to-find resource that does not require fermentation or specialized preparation. Additionally, for potential topical applications, LJ offers superior sensory acceptability compared to acetic acid, which could positively influence patient or farmer compliance.

Future studies will be needed to design formulations that optimize mucosal residence time and tolerability.

## 5. Conclusions

In conclusion, this study provides the first scientific evidence of the antiviral and virucidal activity of LJ against CpHV-1, highlighting its pH-dependent mechanism. While the high acidity of pure juice limits its direct mucosal application due to potential irritation, these results propose LJ as a promising low-cost control strategy for this disease in livestock settings and also offer valuable insights for applied research in the field of human herpesvirus infections.

## Data Availability

All data generated in this study are available in the present article.
